# Reproducibility, responsiveness and validation of the Tampa Scale for Kinesiophobia in patients with ACL injuries

**DOI:** 10.1186/s12955-019-1217-7

**Published:** 2019-09-11

**Authors:** Hui Huang, Masashi Nagao, Hitoshi Arita, Jun Shiozawa, Hirofumi Nishio, Yohei Kobayashi, Haruka Kaneko, Masataka Nagayama, Yoshitomo Saita, Muneaki Ishijima, Yuji Takazawa, Hiroshi Ikeda, Kazuo Kaneko

**Affiliations:** 1 Department of Orthopaedic Surgery, Juntendo University School of Medicine, 2-1-1 Hongo Bunkyo-ku, Tokyo, 113-8421 Japan; 2 Medical Technology Innovation Center, Juntendo University, Tokyo, Japan; 3 Clinical Research & Trial Center, Juntendo University, Tokyo, Japan; 4 Department of Emergency and Critical Care Medicine, Juntendo University Hospital, Tokyo, Japan; 5 Department of Sports Medicine, School of Health & Sports Science, Juntendo University, Chiba, Japan

**Keywords:** Validation study, Tampa Scale for Kinesiophobia (TSK), Anterior cruciate ligament (ACL), The consensus-based standards for the selection of health status measurement INstruments (COSMIN)

## Abstract

**Background and purpose:**

Psychological factors including fear of pain, re-injury during movement (kinesiophbia) affect return-to-sport rates after anterior cruciate ligament (ACL) reconstructive surgery. Clinicians often encounter in the daily practice that athletes explain lack of self-confidence or psychological readiness during the sports activity. The Tampa Scale for Kinesiophobia (TSK) has been used to evaluate psychological outcomes in patients with ACL injuries in many countries and translated into Japanese version in 2013. However, no researchers validated its reliability, validity, and responsiveness of TSK for patients with ACL injury up to now. The purpose of this study was to evaluate the measurement properties of the Japanese version of the TSK (TSK-J) in patients with ACL injuries.

**Study design:**

Cohort study (Diagnostic); Level of evidence, 2.

**Methods:**

This prospective study was performed in the department of orthopaedic surgery at the university hospital of Juntendo from Sep 2016 and Apr 2017. Patients who diagnosed with ACL injury with or without reconstruction surgery completed several patient-reported outcome measures (PROMs) were included in this study. The COnsensus-based Standards for the selection of health status Measurement INstruments (COSMIN) guidelines were used to evaluate reliability, validity, responsiveness, and interpretability of the TSK-J.

**Results:**

222 patients were included in this study. The TSK-J for ACL injured patients showed good internal consistency (Cronbach’s alpha = 0.79) and excellent test-retest reliability (intra-class correlation coefficient, ICC_2,1_ = 0.90, 95% CI = 0.81 to 0.95). In addtion, the TSK-J was significantly but moderately correlated with the IKDC-SKF (*r* = − 0.49, *P* <0.001), VAS-Sports (*r* = − 0.48, *P* <0.001), and JACL-25 (*r* = 0.48, *P* <0.001). The effect size (ES) was small with the Cohen’s d = − 0.2. The minimal important difference (MID) was − 1.3 points. No significant TSK-J score change was observed over 1-year after ACL reconstruction (*r* = − 0.12, *P* <0.001). There were no floor or ceiling effects.

**Conclusions:**

Our study demonstrated that the Japanese version of TSK has good reliability. However, its low validity and responsiveness indicate that it may not the best way to assess psychological factors for patients with ACL injury.

## Introduction

Anterior cruciate ligament (ACL) tear is one of the most common injuries in the young and active population with an estimate of 20,000–30,000 tears and over 15,000 ACL reconstruction procedures performed per year in Japan [1]. Reconstruction surgery is necessary if patients wish to participate in sports unrestrictedly and return to their pre-injury level. However, a recent meta-analysis of return-to-sport outcomes demonstrated that while 85% of patients returned to some form of sports participation after surgery, only 64% returned to their pre-injury level. In addition, only 56% were able to return to competitive sports [2]. Patients with ACL reconstruction may not return to their pre-injury sports or level for a variety of reasons. A multicenter cohort study reported that of the non-returners in this study, 50% cited fear of re-injury as a reason for not returning [3]. A concept of ‘psychological readiness’ following ACL injury has been highlighted as an important factor during sports activity [4].

To assess psychological factors in patients with ACL injury, several measurement instruments were developed and validated [5, 6]. Among those, the Tampa Scale for Kinesiophobia (TSK) has been used to evaluate fear of re-injury, pain or movement in ACL injury patients [7, 8]. Kinesiophobia, which has been defined as an excessive, irrational and debilitating fear of physical movement and activity resulting from a feeling of vulnerability to painful injury or re-injury [9]. According to the past study, the not return to sports group has higher mean scores of TSK-11 than return to sports group after ACL reconstruction surgery [7]. Although the original English version of TSK was translated into Japanese in 2013 [10–12], it has not been validated to be applied to patients with ACL injury.

## Purpose

The purpose of this study was to evaluate the Japanese version of TSK in patients with ACL injury according to the COSMIN checklist.

## Materials and methods

### Participants

This prospective study was performed at the Juntendo university hospital from Sep 2016 to Apr 2017. Patients with the following criteria were included in this study: (1) diagnosed with ACL injury by physical examination and MRI, (2) understand Japanese language, (3) completed the TSK-J, the IKDC Subjective Knee Form (IKDC-SKF), the JACL-25, the Visual Analog Scale for Sports (VAS-Sports/ 0-100 mm) and the Patient Global Impression of Change (PGIC/ 1–7 points), (4) age between 16 and 65 years, and (5) with no mental illness.

### Evaluation of measurement properties

The *TSK* contains 17 items related to pain, fear of movement and re-injury. The score ranges from 17 to 68, and the higher scores, the greater pain, fear of movement and re-injury [13]. Translation and cultural adaptation of the Japanese TSK was performed according to the Principles of Good Practice approach, which is allowing for different ways to achieve the same goal for each step in the process of translation [11, 14].

The *IKDC-SKF* has been developed to assess knee conditions, including symptoms, functions, and sports activities for patients with a variety of knee problems [15]. It consists of 19 items with a range from 0 to 100, and the higher score indicates fewer symptoms, better functions, and higher sports activities. It has been widely used to assess physical factors after ACL reconstruction surgery in many countries [16, 17].

The *JACL-25* was developed and validated to assess fear of motion during daily activity and sports participation for patients with ACL injuries [6]. It contains 25 items with the scores range from 0 to 100, and a higher score indicates a worse condition. Each item in JACL-25 was defined with specific life-experiences (knee instability condition) that may frequently occur in patients with ACL injury.

The *PGIC* reflects patients who make a subjective judgment about the meaning of change (improvement) following treatment. It is answered on a 7-point scale of 1 = very much worse; 2 = much worse; 3 = minimally worse; 4 = no change; 5 = minimally improved; 6 = much improved; 7 = very much improved.

### Measurement properties

We evaluated the reliability, validity, responsiveness, and interpretability to the TSK-J according to the COSMIN guidelines. In addition, the quality of the TSK-J was also evaluated by current updated criteria for good measurement properties [18].

### Reliability

This domain contains three measurement properties, i.e., internal consistency, test-retest reliability, and measurement error [19]. Internal consistency is considered as a measure of scale reliability and evaluates how closely related a set of items are as a group. Also, test-retest reliability is the closeness of the agreement between the results of successive measurements of the same measurand carried out under the same conditions of measurement. To avoid testing patients with the unstable condition or occurring recall bias, the re-test was performed after 4 weeks after the primary test. At last, the measurement error was calculated by using the standard error of measurement (SEM).

### Validity

This domain also contains three measurement properties, i.e., content validity, criterion validity and construct validity [20]. Content validity mainly examines the measurement aim, the target population and the concepts of the questionnaire. It should provide researchers or clinicians to select item related to the target population. Criterion validity was evaluated by calculating the correlation between TSK-J and IKDC-SKF, which is widely used as a “gold” standard instrument. Construct validity was assessed by testing predefined specific hypotheses; that is, how many results are in accordance with predefined hypotheses.

1. The TSK-J scores will have a strong positive correlation with the JACL-25 scores.
2. Patients who answer the PGIC scale to “improved (including minimally improved, much improved, and very much improved)” will have a lower TSK-J mean score than those who answer to “no change”.
3. The TSK-J scores will have a strong negative correlation with the VAS-Sports scores.
4. The TSK-J scores will demonstrate a strong negative correlation with the following times after surgical treatment.

It has been suggested that hypotheses are specified in advance, and at least 75% of the results are in correspondence with these hypotheses. Also, the correlation coefficient could be considered in five degrees of very strong (*r* = 0.80 to 1.00), strong (*r* = 0.60 to 0.79), moderate (*r* = 0.40 to 0.59), weak (*r* = 0.20 to 0.39), and very weak (*r* = 0.00 to 0.19), respectively.

### Responsiveness

Responsiveness has been defined as the ability of a questionnaire to detect clinically important change over time in the construct to be measured [19]. We calculated the change scores of the TSK-J between baseline (pre-surgery) and post-surgery with the time interval of 10 weeks.

### Interpretability

Floor or ceiling effect was defined as more than 15% of participants reported the minimum or maximum scores, respectively. In addition, the smallest detectable change for individual changes (SDC_ind_) and the group change of SDC_gro_ were also calculated. Minimal important change (MIC) of Within-group was measured with a mean score change who reported the PGIC scale as “minimally improved” at repeat time (14 weeks) according to an anchor-based approach. Additionally, the minimal important difference (MID) was calculated by the mean score change between “minimally improved” and “no change” group [21].

### Statistical analysis

All analyses were performed with the R-studio Software (R-Studio, Inc., Boston, USA).

Good measurement properties were defined by using the updated criteria for COSMIN guideline [18]. Internal consistency was calculated with Cronbach alpha and had been deemed to be sufficient if it is ≥0.70. The test-retest reliability was calculated by using the intra-class correlation coefficient (ICC_2,1_) and recommended as a minimum standard for reliability if it is greater than 0.7. The measurement error was calculated by using the ANOVA analysis. Pearson or Spearman correlation coefficients were used to calculating the correlation with gold standard and analysis of the discriminative hypothesis. Correlation with the gold standard was sufficient if the *r* ≥ 0.70. Responsiveness was calculated both the Cohen’s *d* and the receiver operating characteristics (ROC) curve (AUC) and at least 0.70 of AUC to be sufficient. At last, the SDC_ind_ and SDC_gro_ were calculated according to the formula of SDC_ind_ = 1.96 * √2 *SEM and SDC_gro_ = SDC_ind_ /√n, respectively [22].

## Results

222 of 255 the patients included in this study. 33 patients excluded from this study due to: patients who did not complete either of the questionnaires = 18, did not answer more than two items in the IKDC-SKF =7, and under 16-year old =8. Their demographics were presented as below (Table 1).

**Table 1:** Demographics of participants

Characteristics	
Age, mean (SD), year	32.4 (12.4)
Gender, n (%)	
Male	141 (63.5)
Female	81 (36.5)
BMI, mean (SD)	24.6 (4.4)
Post-operative, n (%)	184 (82.9)
Non-operative, n (%)	38 (17.1)
Side, n (%)	
Left	103 (46.4)
Right	100 (45.0)
Bilateral	19 (8.6)
Injury mechanism, n (%)	
Contact	44 (17.5)
Noncontact	153 (60.7)
Unclear	55 (21.8)
Concomitant Injury, n (%)	
Meniscus	120 (64.2)
Articular cartilage	55 (29.4)
Other ligaments	12 (6.4)

*SD* standard deviation, *BMI* body mass index

### Missing data

222 patients completed a total of 350 times in this study. Of this, 8 patients did not answer 1 or more items of the TSK-J instrument. The amount of missing data was 0.47% of the 17 items in TSK-J.

### Reliability

The results of the internal consistency, test-retest reliability and measurement error for the TSK-J were listed in Table 2.

**Table 2:** Measurement properties of reliability

Measurement properties	TSK-J
Cronbach’s alpha, *n* = 222 (95% CI)	0.79 (0.76 to 0.83)
ICC _2,1_, *n* = 43 (95% CI)	0.90 (0.81 to 0.95)
Baseline score, Mean (SD)	39.7 (6.75)
Retest score, Mean (SD)	38.4 (6.11)
*P* value	0.3426
95% CI	−1.44 to 4.09
SEM, *n* = 43	2.75

*TSK-J* Japanese Tampa Scale for Kinesiophobia, *CI* confidence interval, *ICC* intra-class correlation coefficient, *SD* standard deviation, *SEM* Standard error of measurement

Internal consistency of the TSK-J was good, with the Cronbach’s alpha (95% CI) of 0.79 (0.76 to 0.83).

Also, test-retest reliability was excellent with the ICC _2,1_ of 0.90 (0.81 to 0.95) (Time interval, days ± SD = 28.77 ± 8.3) (*n* = 43).

The measurement error of the SEM for the TSK-J was 2.75.

### Validity

#### Content validity

The content validity of TSK-J was presented below (Table 3).

**Table 3:** Content validity of the TSK-J

Characteristics	
Measurement aim	Quantifying pain, fear avoidance, fear of re-injury during movement because of a previous injury [23].
Target population	The target population is unclear but has been widely used for low back pain and any other chronic pain [24, 25].
Concept	TSK has commonly been used for patients with ACL injury to evaluate psychological factors.

*TSK-J* Japanese Tampa Scale for Kinesiophobia, *ACL* anterior cruciate ligament

#### Criterion validity

The criterion validity of the TSK-J between the IKDC-SKF resulted in a moderate but significant correlation coefficient (*r* < − 0.49, *P* < 0.001 in Table 4).

**Table 4:** Correlation between the TSK-J and other outcomes (*n* = 222)

	IKDC-SKF	VAS-Sports	JACL-25	*P*-value
TSK-J, *r*	−0.49	− 0.48	0.48	< 0.001
IKDC-SKF, *r*	–	0.80	−0.87	< 0.001
JACL-25, *r*	−0.87	−0.82	–	< 0.001

*TSK-J* Japanese Tampa Scale for Kinesiophobia, *IKDC-SKF* International Knee Documentation Committee Subjective Knee Form, *VAS-Sports* Visual Analog Scale for Sports, *JACL-25* Japanese Anterior Cruciate Ligament 25

#### Construct validity

**Fig. 1:**
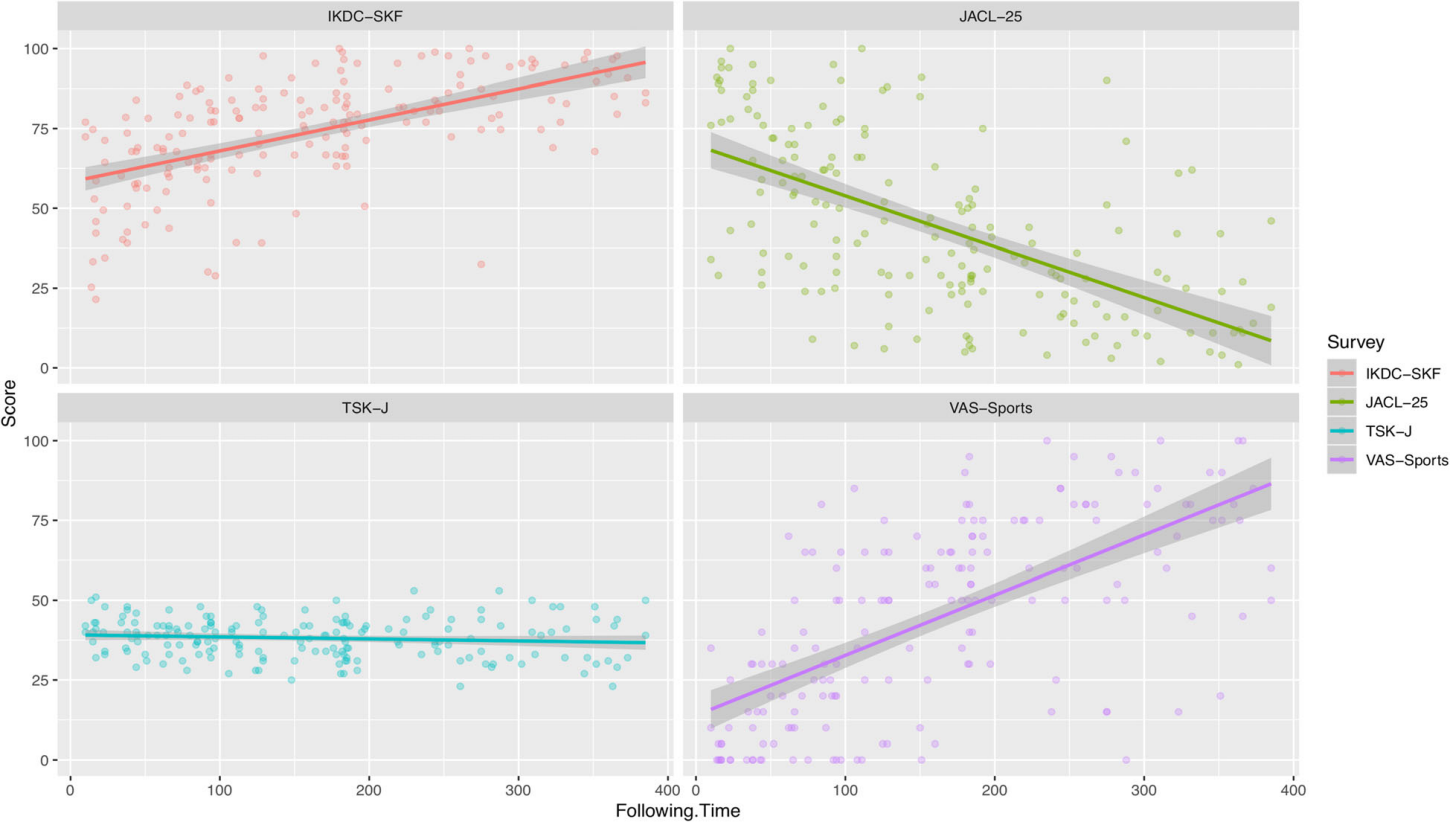
Correlation between TSK-J and following time. Almost no change found in the TSK-J following time (*r* = − 0.12)

1. The TSK-J had a moderate positive correlation with the JACL-25 (*r* = 0.48) (Table 4)
2. Patients who answered the PGIC scale to “improved (including minimally improved, much improved, and very much improved)” had a lower TSK-J mean score than those who answered to “no change” (“improved” = − 0.7, “no change” = 0.5, Table 5).
3. The TSK-J had a moderate negative correlation with the VAS-Sports (*r* = − 0.48) (Table 4)
4. The TSK-J scores had no change until about 400 days after ACL reconstruction surgery (*r* = − 0.12) (Fig. 1, Table 5)

**Table 5:** Responsiveness

	TSK-J	IKDC-SKF	JACL-25	
Pre-surgery, mean (SD), *n* = 18	39.3 (8.6)	65.7 (16.7)	64.1 (25.6)	
Post-surgery, mean (SD), *n* = 18	38.1 (4.9)	70.6 (13.8)	51.0 (24.2)	
ES^a^, Cohen’s d (95% CI), *n* = 18	0.2 (−0.50 to 0.85)	0.3 (−0.36 to 1.00)	0.5 (− 0.16 to 1.21)	
Following Time, *r*, *n* = 97	−0.12	0.62	−0.62	
AUC, *n* = 72	0.54	0.61	0.7	
*P* Value	NS	0.004	0.015	

*SD* standard deviation, *TSK-J* Japanese Tampa Scale for Kinesiophobia, *IKDC-SKF* International Knee Documentation Committee Subjective Knee Form, *JACL-25* Japanese Anterior Cruciate Ligament 25, *AUC* Area under the curve, *NS* no significant difference, *ES* effect size

^a^Cohen’s d was calculated by the mean time interval of 10 weeks

### Responsiveness

**Fig. 2:**
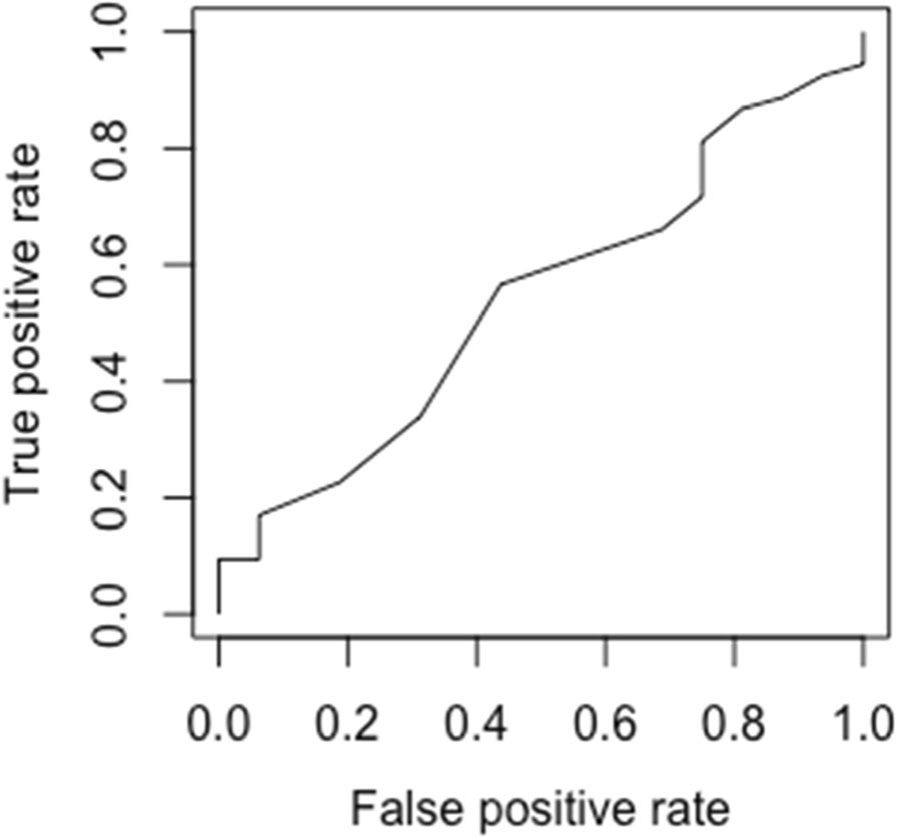
ROC between “no change” and “improved” (*n* = 72). ROC, receiver operating characteristics. The area under the curve (AUC) between “no change” and “improved” demonstrate fail accuracy of AUC = 0.54

The ES of Cohen’s d was − 0.2 (small effect size), and the correlation between the TSK-J and following time was − 0.12 (Table 5, Fig. 1) The AUC for the TSK-J was 0.54, (Fig. 2, Table 5) and *P*-value of AUC of the TSK-J shows no significant difference (*P* > 0.05).

### Interpretability

There were no floor or ceiling effects in the TSK-J scale. The SDC for the TSK-J scale was 7.6 for individuals, and 1.2 for groups. The MIC and MID were − 0.8 and − 1.3, respectively (Table 6).

**Table 6:** Interpretability

Characteristic	n	TSK-J
PGIC, mean change (SD)		
Very much worse	–	–
Much worse	–	–
Minimally worse	4	− 2.5 (1.7)
No change	17	0.5 (5.6)
Minimally improved	16	−0.8 (4.3)
Much improved	29	0.4 (6.7)
Very much improved	10	−3.8 (5.8)
Baseline mean (SD)	76	39.3 (7.3)
Repeat mean (SD)	76	38.8 (7.0)
MIC	16	−0.8
MID	33	−1.3
SDC_ind_	43	7.6
SDC_gro_	43	1.2
Ceiling effect (%)	222	0
Floor effect (%)	222	0

*PGIC* patient global impression of change, *SDC*_*ind*_ smallest detectable change for individual changes, *SDC*_*gro*_ smallest detectable change for group, *SD* standard deviation, *MIC* minimal important change, *MID* minimal important difference, *TSK-J* Japanese Tampa Scale for Kinesiophobia

## Discussion

This is the first study to assess the validity, reliability, and responsiveness of the TSK for patients with ACL injury according to the COSMIN checklist. The internal consistency and test-retest reliability resulted in good reliability. In the validity domain, the content validity was interpretable, the criterion validity between the TSK-J and the IKDC-SKF resulted in a moderate correlation coefficient, which is lower compared to the IKDC-SKF and the JACL-25 (Table 4) Only one of four hypotheses (No. 2) in the construct validity domain was in accordance with the hypothesis. Furthermore, the responsiveness of TSK-J resulted in low rating and very weak time-dependent change (Fig. 1). The MID for the TSK was − 1.3, which means that change as large as 1.3, it may be important for patients. There were no floor or ceiling effects.

According to the past validation study, only the internal consistency of TSK-11 has been validated for patients with ACL injury [13], besides that, other measurement properties of validity and responsiveness were unknown in both the TSK and TSK-11. In this present study, the data indicated a low rating of the responsiveness by calculating the change scores (ES of the Cohen’d = 0.2) of the TSK-J between before and post-surgery with the time interval of 10 weeks and very weak correlation of time-dependent change (*r* = − 0.12 following post-surgery 1-year). Compared to the IKDC-SKF and the JACL-25, both results of validity and responsiveness indicated insufficient rating (Tables 4 and 5) However, one study reported that the TSK-11 scores after ACL-reconstruction surgery continued to decrease through 12 weeks and significantly different from baseline [8]. Another study also reported that not return to sports group has higher mean scores of TSK-11 than return to the sports group at both 6 months and 1 year after ACL reconstruction [7].To find out which factors lead to this gap, we also calculated the correlation between each item scores and TSK total scores (Table 7), found that only 2 of 17 items have good correlations (*r* > 0.7) and 5 items lower than 0.5. This result indicated that the item 4, 5, 8, 16, and 17 might not suit the patients with ACL injury because of the low correlation. This data could affect the result of the validity and the responsiveness. For another factor, we speculate that the cultural difference between Asians and Westerners may show different results when testing psychological factors. It has been argued that there are significant psychological differences between East Asians and Westerners that are rooted in long-standing differences between East Asian and Western civilisations [26]. The attentional differences were further presented to be an important factor contributing to cultural differences between Japanese and American in higher cognitive mechanisms [27]. Japanese has unique characteristics in response questions. For example, they did not like directly to reject someone or something, but response soft or reject indirectly. We noticed that patients in this study intended to answer the middle answer (“disagree” or “agree”) than extreme answer (“strongly disagree” or “strongly agree”).

**Table 7:** Correlation between each item (*n* = 222)

Item No.	Correlation between TSK-J total score, *r*
Q01	0.68
Q02	0.64
Q03	0.72
Q04	0.27
Q05	0.42
Q06	0.59
Q07	0.61
Q08	0.20
Q09	0.69
Q10	0.61
Q11	0.67
Q12	0.57
Q13	0.50
Q14	0.71
Q15	0.66
Q16	0.05*
Q17	0.38

*TSK-J* Japanese Tampa Scale for Kinesiophobia

*P > 0.05

Further study was needed to edit or adapt some content of TSK-J items to obtain more appropriate scale (remove weak correlation items or edit them more correlatively) which may help Japanese clinician assess kinesiophobia more exactly.

Psychological factors have been significantly associated with returning to the preinjury activity. There are several questionnaires has been applied to evaluate psychological readiness for patients after ACL surgery. One of the common scales, the ACL-RSI has been translated and validated to evaluate psychological readiness to resume sport after ACL reconstruction in many countries [28–31]. However, the Japanese version of the ACL-RSI has not been validated and not contain psychological factors of fear of pain during movement. Therefore, we did not use it for the study. The TSK also has been used to evaluate the psychological factors of kinesiophobia for patients with ACL injury.

This study has a limitation. As the measurement properties, some of their sample size in this study may not be sufficient (ICC, SEM, and MID et al. *n* < 50), despite the criteria for measurement properties that positive rating should be with a sample size of at least 50 patients to be considered [32].

## Conclusion

The TSK-J has good reliability for assessing patients with ACL injury. However, its low validity and responsiveness indicate that it is not the best patient-reported outcome measure for psychological factors for patients with ACL injury.

## Data Availability

The datasets used and/or analysed during the current study are available from the corresponding author on reasonable request.

## Abbreviations

ACL: Anterior cruciate ligament
AUC: Area under the curve
BMI: Body Mass Index
CI: Confidence interval
COSMIN: COnsensus-based Standards for the selection of health status Measurement Instruments
ICC: Intra-class correlation coefficient
IKDC-SKF: International Knee Documentation Committee Subjective Knee Form
JACL-25: Japanese Anterior Cruciate Ligament questionnaire 25
MIC: Minimal important change
MID: Minimal important difference
PGIC: Patients’ Global Impression of Change
PROMs: patient-reported outcome measures
ROC: Receiver operating characteristics
SD: Standard deviation
SDC_gro_: Smallest detectable change for group changes
SDC_ind_: Smallest detectable change for individual changes
SEM: Standard error of measurement
TSK: Tampa Scale for Kinesiophobia
TSK-J: Japanese version of the TSK
VAS-Sports: Visual Analog Scale for Sports

## References

[1] Japanese Orthopaedic Society of Knee AaSM, The Japanese Orthopaedic Association (2012). Japanese Orthopaedic Association (JOA) Clinical Practice Guideline on the management of Anterior Cruciate Ligament Injury of the Knee.

[2] Ardern CL, Webster KE, Taylor NF, Feller JA (2011). Return to sport following anterior cruciate ligament reconstruction surgery: a systematic review and meta-analysis of the state of play. Br J Sports Med.

[3] McCullough KA, Phelps KD, Spindler KP, Matava MJ, Dunn WR, Parker RD, Reinke EK, Group M (2012). Return to high school- and college-level football after anterior cruciate ligament reconstruction: a multicenter Orthopaedic outcomes network (MOON) cohort study. Am J Sports Med.

[4] Ardern CL, Osterberg A, Tagesson S, Gauffin H, Webster KE, Kvist J (2014). The impact of psychological readiness to return to sport and recreational activities after anterior cruciate ligament reconstruction. Br J Sports Med.

[5] Webster KE, Feller JA, Lambros C (2008). Development and preliminary validation of a scale to measure the psychological impact of returning to sport following anterior cruciate ligament reconstruction surgery. Phys Ther Sport.

[6] Nagao M, Doi T, Saita Y, Kobayashi Y, Kubota M, Kaneko H, Takazawa Y, Ishijima M, Kurosawa H, Kaneko K (2016). A novel patient-reported outcome measure for anterior cruciate ligament injury: evaluating the reliability, validity, and responsiveness of Japanese anterior cruciate ligament questionnaire 25. Knee Surg Sports Traumatol Arthrosc.

[7] Lentz TA, Zeppieri G, George SZ, Tillman SM, Moser MW, Farmer KW, Chmielewski TL (2015). Comparison of physical impairment, functional, and psychosocial measures based on fear of reinjury/lack of confidence and return-to-sport status after ACL reconstruction. Am J Sports Med.

[8] Chmielewski TL, Zeppieri G, Lentz TA, Tillman SM, Moser MW, Indelicato PA, George SZ (2011). Longitudinal changes in psychosocial factors and their association with knee pain and function after anterior cruciate ligament reconstruction. Phys Ther.

[9] Nijs J, De Meirleir K, Duquet W (2004). Kinesiophobia in chronic fatigue syndrome: assessment and associations with disability. Arch Phys Med Rehabil.

[10] Nishigami T, Mibu A, Tanaka K, Yamashita Y, Watanabe A, Tanabe A (2017). Psychometric properties of the Japanese version of short forms of the pain catastrophizing scale in participants with musculoskeletal pain: a cross-sectional study. J Orthop Sci.

[11] Kikuchi N, Matsudaira K, Sawada T, Oka H (2015). Psychometric properties of the Japanese version of the Tampa Scale for Kinesiophobia (TSK-J) in patients with whiplash neck injury pain and/or low back pain. J Orthop Sci.

[12] Matsudaira K, Inuzuka K, Kikuchi N, Sakae C, Arisaka M, Isomura T, Miller RP (2012). Development of a Japanese Version of the Tampa Scale for Kinesiophobia (TSK-J) : Translation and Linguistic Validation. J Musculoskelet Pain Res.

[13] George SZ, Lentz TA, Zeppieri G, Lee D, Chmielewski TL (2012). Analysis of shortened versions of the Tampa Scale for Kinesiophobia and pain catastrophizing scale for patients after anterior cruciate ligament reconstruction. Clin J Pain.

[14] Wild D, Grove A, Martin M, Eremenco S, McElroy S, Verjee-Lorenz A, Erikson P (2005). Translation ITFf, cultural a: principles of good practice for the translation and cultural adaptation process for patient-reported outcomes (PRO) measures: report of the ISPOR task force for translation and cultural adaptation. Value Health.

[15] Irrgang JJ, Anderson AF, Boland AL, Harner CD, Kurosaka M, Neyret P, Richmond JC, Shelborne KD (2001). Development and validation of the international knee documentation committee subjective knee form. Am J Sports Med.

[16] Sundemo D, Sernert N, Kartus J, Hamrin Senorski E, Svantesson E, Karlsson J, Samuelsson K (2018). Increased postoperative manual knee laxity at 2 years results in inferior long-term subjective outcome after anterior cruciate ligament reconstruction. Am J Sports Med.

[17] Sonnery-Cottet B, Saithna A, Cavalier M, Kajetanek C, Temponi EF, Daggett M, Helito CP, Thaunat M (2017). Anterolateral ligament reconstruction is associated with significantly reduced ACL graft rupture rates at a minimum follow-up of 2 years: a prospective comparative study of 502 patients from the SANTI study group. Am J Sports Med.

[18] Prinsen CAC, Mokkink LB, Bouter LM, Alonso J, Patrick DL, de Vet HCW, Terwee CB (2018). COSMIN guideline for systematic reviews of patient-reported outcome measures. Qual Life Res.

[19] Mokkink LB, Terwee CB, Patrick DL, Alonso J, Stratford PW, Knol DL, Bouter LM, de Vet HC (2010). The COSMIN study reached international consensus on taxonomy, terminology, and definitions of measurement properties for health-related patient-reported outcomes. J Clin Epidemiol.

[20] Mokkink LB, Terwee CB, Patrick DL, Alonso J, Stratford PW, Knol DL, Bouter LM, de Vet HC (2010). The COSMIN checklist for assessing the methodological quality of studies on measurement properties of health status measurement instruments: an international Delphi study. Qual Life Res.

[21] Beard DJ, Harris K, Dawson J, Doll H, Murray DW, Carr AJ, Price AJ (2015). Meaningful changes for the Oxford hip and knee scores after joint replacement surgery. J Clin Epidemiol.

[22] de Boer MR, de Vet HC, Terwee CB, Moll AC, Volker-Dieben HJ, van Rens GH (2005). Changes to the subscales of two vision-related quality of life questionnaires are proposed. J Clin Epidemiol.

[23] Miller RP, Kori SH, Todd DD (1991). The Tampa scale: a measure of Kinisophobia. Clin J Pain.

[24] HajGhanbari B, Holsti L, Road JD, Darlene Reid W (2012). Pain in people with chronic obstructive pulmonary disease (COPD). Respir Med.

[25] Crombez G, Vlaeyen JW, Heuts PH, Lysens R (1999). Pain-related fear is more disabling than pain itself: evidence on the role of pain-related fear in chronic back pain disability. Pain.

[26] Nisbett RE, Peng K, Choi I, Norenzayan A (2001). Culture and systems of thought: holistic versus analytic cognition. Psychol Rev.

[27] Masuda T, Nisbett RE (2001). Attending holistically versus analytically: comparing the context sensitivity of Japanese and Americans. J Pers Soc Psychol.

[28] Bohu Y, Klouche S, Lefevre N, Webster K, Herman S (2015). Translation, cross-cultural adaptation and validation of the French version of the anterior cruciate ligament-return to sport after injury (ACL-RSI) scale. Knee Surg Sports Traumatol Arthrosc.

[29] Chen T, Zhang P, Li Y, Webster K, Zhang J, Yao W, Yin Y, Ai C, Chen S (2017). Translation, cultural adaptation and validation of simplified Chinese version of the anterior cruciate ligament return to sport after injury (ACL-RSI) scale. PLoS One.

[30] Harput G, Tok D, Ulusoy B, Eraslan L, Yildiz TI, Turgut E, Demirci S, Duzgun I, Tunay VB, Baltaci G, Ergun N (2017). Translation and cross-cultural adaptation of the anterior cruciate ligament-return to sport after injury (ACL-RSI) scale into Turkish. Knee Surg Sports Traumatol Arthrosc.

[31] Sala-Barat E, Alvarez-Diaz P, Alentorn-Geli E, Webster KE, Cugat R, Tomas-Sabado J. Translation, cross-cultural adaptation, validation, and measurement properties of the Spanish version of the anterior cruciate ligament-return to sport after injury (ACL-RSI-Sp) scale. Knee Surg Sports Traumatol Arthrosc. 2019. 10.1007/s00167-019-05517-z. 10.1007/s00167-019-05517-z 31089791

[32] Terwee CB, Bot SD, de Boer MR, van der Windt DA, Knol DL, Dekker J, Bouter LM, de Vet HC (2007). Quality criteria were proposed for measurement properties of health status questionnaires. J Clin Epidemiol.

